# A 3D Multiscale Model to Explore the Role of EGFR Overexpression in Tumourigenesis

**DOI:** 10.1007/s11538-019-00607-y

**Published:** 2019-04-23

**Authors:** Anass Bouchnita, Stefan Hellander, Andreas Hellander

**Affiliations:** Division of Scientific Computing, Department of Information Technology, Uppsala University, 75105 Uppsala, Sweden

**Keywords:** EGFR, Tumour growth, Agent-based modelling, Brownian Dynamics

## Abstract

The epidermal growth factor receptor (EGFR) signalling cascade is one of the main pathways that regulate the survival and division of mammalian cells. It is also one of the most altered transduction pathways in cancer. Acquired mutations in the EGFR/ERK pathway can cause the overexpression of EGFR on the surface of the cell, while others downregulate the inactivation of switched on intracellular proteins such as Ras and Raf. This upregulates the activity of ERK and promotes cell division. We develop a 3D multiscale model to explore the role of EGFR overexpression on tumour initiation. In this model, cells are described as individual objects that move, interact, divide, proliferate, and die by apoptosis. We use Brownian Dynamics to describe the extracellular and intracellular regulations of cells as well as the spatial and stochastic effects influencing them. The fate of each cell depends on the number of active transcription factors in the nucleus. We use numerical simulations to investigate the individual and combined effects of mutations on the intracellular regulation of individual cells. Next, we show that the distance between active receptors increase the level of EGFR/ERK signalling. We demonstrate the usefulness of the model by quantifying the impact of mutational alterations in the EGFR/ERK pathway on the growth rate of in silico tumours.

## Introduction

The EGFR/ERK cascade is one of the main mitogen activated-protein kinase (MAPK) pathways that regulate the survival and division of several mammalian cells (Orton et al. 2005). This signalling cascade is stimulated when epidermal growth factors (EGFs) bind to their receptors (EGFRs) present on the membrane surface of cells. As a result, Ras proteins, which diffuse in proximity of the corresponding G-protein site, bind to GTP molecules and switch to the Ras-GTP active state. The diffusing Ras-GTPs subsequently interact with Raf which, upon activation, dually phosphorylate and activate MEK proteins. Subsequently, MEKs participate in the formation of active ERKs. Then, ERK proteins phosphorylates RSK and both of them translocate to the nucleus where they activate several transcription factors such as CREB, Fos, and Elk-1.

In cancer, acquired mutations disrupt the normal functioning of the EGFR/ERK pathway. Some of these mutations lead to the upregulation of ERK activation which promotes the survival and proliferation of the cell. The most clinically observed mutations of the EGFR/ERK pathway concern changes in the Ras and Raf oncogenes as well EGFR. In this context, it was observed that 30% of human cancers contain a mutation in the Ras gene (K-Ras, H-Ras, N-Ras) (Bos 1989). These mutations prevent the automatic switching off of the Ras protein upon its activation. Another type of mutation that is often observed in human cancers concerns the B-Raf oncogene (Davies et al. 2002). This mutation leads to the production of permanently activated Raf proteins.

Alterations in the EGFR gene represent another type of the commonly observed cancerous mutations. These changes can provoke the overexpression of EGFR proteins by the cell. EGFR mutations are usually observed in lung cancer as more than 60% of the non-small lung carcinoma cells overexpress EGFRs (da Cunha Santos et al. 2011). There exist several cancer treatments that target the different components of the EGFR/ERK pathway (Roberts and Der 2007). For example, EGFR inhibitors are a class of these treatments that directly block the epidermal growth factor receptors and prevent their activation. Other drugs used in cancer treatment infiltrate the tumour cells and inhibit the activation of some proteins in the EGFR/ERK cascade. Overall, a proper understanding of the effects of mutations on cells is of paramount importance to design more effective anti-cancer therapeutic strategies.

Due to its importance, the EGFR/ERK pathway has been extensively studied experimentally and computationally in previous works. The dynamics of the EGF-receptor were studied using a variety of modelling techniques (Wiley et al. 2003). Detailed simulation of the EGFR/ERK cascade signalling was possible using ordinary differential equations (ODEs). For example, one of the developed models that describe the EGFR/ERK pathway consists of 94 state variables and 95 parameters (Schoeberl et al. 2002). Other ODE-based models quantified the effects of cancerous mutations on the kinetics of the EGFR/ERK cascade upon the activation of EGF receptors (Brown et al. 2004; Orton et al. 2009).

Several modelling techniques and methods were previously used to describe tumour development. The evolution of the populations of tumour cells were often represented using a continuous approach. In this context, both ordinary differential equations and partial differential equations (PDEs) were used to build mathematical models that study several questions related to the organization of tumours (Byrne and Chaplain 1996; Glass 1973; Wise et al. 2008). For example, it was possible to use continuous models to investigate other important questions in oncology such as the intraclonal heterogeneity of tumours (Stiehl et al. 2014; Walenda et al. 2014) and its role in chemotherapy resistance (Panetta 1998). The implementation of continuous models is straightforward, and it is possible to analyse them analytically in order to derive key insights on the underlying mechanisms that govern tumour growth. However, they do not accurately capture cell–cell and/or receptor–ligand interactions. For this reason, discrete models, where cells are represented as individual agents, are often formulated to describe cancer systems. For example, cellular automata (CA) models were previously used to simulate the self-organization of tumour cells (Aubert et al. 2006). CA, as well as the other discrete modelling techniques, describes cells as individual objects that move, interact, divide, and die. Discrete models can be either on-lattice or off-lattice. These models focus on the interactions between cells, but they do not capture the intracellular and extracellular mechanisms underlying the regulation of cell fate. In reality, complex physiological systems, such as cancerous tumours, are regulated by a wide range of processes taking place at different scales of space and time. In this context, multiscale models seek to combine the previously described models in order to benefit from their advantages and overcome their shortcomings.

Hybrid discrete-continuous models, which can be also have a multiscale organization, have been applied successfully to simulate tumour growth (Ramis-Conde et al. 2008, 2009; Zhang et al. 2009). In these models, cells are represented as discrete objects whose intracellular and extracellular regulation are described by continuous models (ODEs and PDEs). We have previously used this modelling technique to describe the development of multiple myeloma, its intraclonal heterogeneity, and effect on erythropoiesis (Bouchnita et al. 2016a, 2017a). Yet, these hybrid models do not capture the spatial and stochastic effects that shape the regulation of cells. Indeed, a key insight from the last decade of systems biology research is that stochastic models of chemical kinetics can capture and predict effects from stochasticity in gene regulation caused by low molecular copy numbers. Recently, a range of studies have highlighted how the spatial dimension of intracellular regulation plays a key role in the functioning of the underlying systems (Elf and Ehrenberg 2004; Fange and Elf 2016; Lawson et al. 2013; Sturrock et al. 2013b, a; van Zon et al. 2006; You et al. 2016).

The majority of the previously developed multiscale models of tumour growth relies on a deterministic description for the intracellular and extracellular regulatory processes to reduce the computational cost. Furthermore, most of them focus on the higher-level physiological processes such as cell–cell and cell–microenvironment interactions. However, acquired cancerous mutations alter the normal functioning of cellular components such as receptors and proteins involved in the regulation of the cell. In this work, we present a novel multiscale model that combines three modules describing physiological processes that regulate tumour growth at different scales. These modules include the interaction between EGFRs and their ligands, the intracellular regulation of individual cells via the EGFR/ERK pathway, and the 3D multicellular biomechanics of early-stage tumour growth. We represent cells as discrete objects that can move, grow, divide, and die by apoptosis. Each cell is modelled as a sphere with a nucleus, a cytoplasm where different molecules diffuse, and a membrane with surface receptors. The interaction between receptors and their extracellular ligands (EGFs) is described with Brownian Dynamics. The same method is used to simulate the dynamics of intracellular regulation. The fate of each cell depends on the number of activated transcription factors in its nucleus. Thus, the model integrates a detailed spatial model of the intracellular regulation following extracellular signalling, and tumour biomechanics. The fidelity of the model makes it suitable to properly study the fine grained aspects of tumour initiation and early-stage growth. We first apply the model to study the effects of acquired mutations in the EGFR/ERK pathway on the single-cell dynamics and quantify the impact of the distance between active EGFRs on the fate of individual cells. Then, we describe the pathogenesis of the 3D in silico tumours and the effect of mutational changes on their growth rate.

The remainder of this paper is organized as follows: Sect. 2 introduces the multiscale model of tumour growth, including the different modules and the techniques used to implement them. Section 3 presents the results of numerical simulations quantifying the effects of mutational alterations on the intracellular regulation of individual cells and their impact on tumourigenesis. The novelty, contributions, and limitations of this study are discussed in Sect. 4.

## A Multiscale Model to Simulate the Effect of EGFR/ERK Signal Transduction on 3D Tumour Growth

**Fig. 1:**
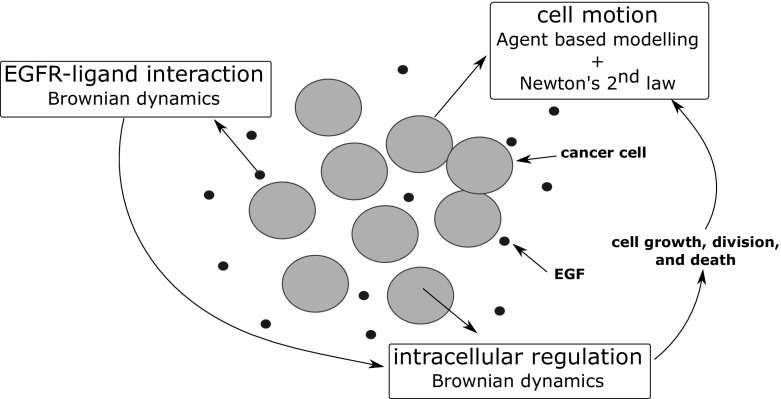
Schematic representation of the multiscale model. Cells are represented as individual soft spheres that can move, divide, and die by apoptosis. The fate of each cell depends on its intracellular regulation described by Brownian Dynamics. The same method is used to capture the interaction between EGFRs and their ligands at the outer surface of the cell

In this work, we develop a multiscale model capable of describing the effects of mutational changes in the EGFR/ERK pathway on tumour initiation. In this model, cells are represented as discrete spheres that move, divide, and die by apoptosis. EGF receptors are present at the membrane of each cell and can bind to their ligands (EGFs). This interaction initiates the EGFR/ERK signalling process in the cell. At the intracellular level, G-protein binding sites activate Ras proteins which results in the induction of the Ras/Raf/MEK/ERK cascade. The final product of this signalling pathway, ERK, translocates to the nucleus and upregulates the expression of downstream transcription factors. These transcription factors play a key role in the regulation of cell fate. In order to describe this signalling process under normal conditions and in cancer, we develop a novel model that integrates three modules for each of the underlying processes: ligand–receptor interactions (Sect. 2.1) , intracellular regulation (Sect. 2.2), and multicellular biomechanics (Sect. 2.3) (Fig. 1). The model contains several sources of noise presented in Sect. 2.4. Details about the computer implementation of the model are provided in “Appendix B”.

### Extracellular Regulation of Cells Through EGFR–Ligand Interactions

**Fig. 2:**
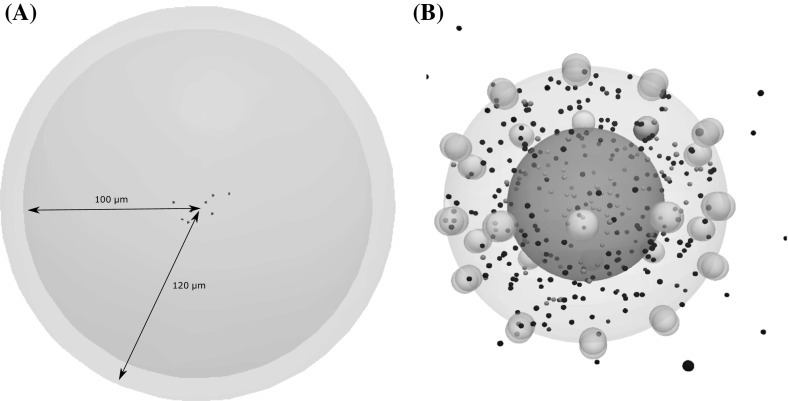
A description of the computational domain and the architecture of individual cells. **a** The computational domain is represented by a sphere with an outer layer where EGF ligands are constantly introduced (light pink). **b** The physiologically relevant structure of an individual cell. The outer cell membrane, as well as inactive receptors, is shown with transparent white, while the nucleus is shown in dark grey. Active receptors are displayed in solid yellow, and intracellular molecules are shown as particles with different colours depending on their type (Color figure online)

We consider a spherical computational domain with a radius of 120 mm. EGFs are introduced periodically at random locations on the outer boundary of this spherical domain (Fig. 2a). They are introduced at the vicinity of the computational domain at a constant rate such that their total number remains equal to approximately 660 EGF in the absence of cells. These proteins diffuse through the extracellular matrix and will be removed from the computational domain upon their degradation. The necessary time for the degradation of each EGF protein is sampled from an exponential distribution. Furthermore, we consider that EGFs reflect if they cross the outer boundary of the computational domain. We assign a reaction radius to each EGF and EGFR protein. EGFs bind reversibly to EGFRs if their reaction radii overlap. As a result, the EGFR becomes activated and induces the intracellular signalling of the cell.

In the present model, we represent each EGFR cluster as two spheres clamped to the cell membrane. The axis that crosses the centres of these two spheres is perpendicular to the tangent of the curve of the cell surface (Fig. 2b). Therefore, one sphere will always remain at the outer of the cell, while the other will be inside the cell. We consider that receptors are uniformly distributed on the surface of each cell. A receptor can exist in two states: ‘on’ and ‘off’. The ‘on’ state represents the active state, while the ‘off’ corresponds to the inactive one. The active receptor will remain in the ‘on’ state for a period of time that corresponds to the residence time. The waiting times for EGF degradation and EGFR–ligand dissociation are sampled from an exponential distribution with a parameter equal to the corresponding reaction rate.

**Fig. 3:**
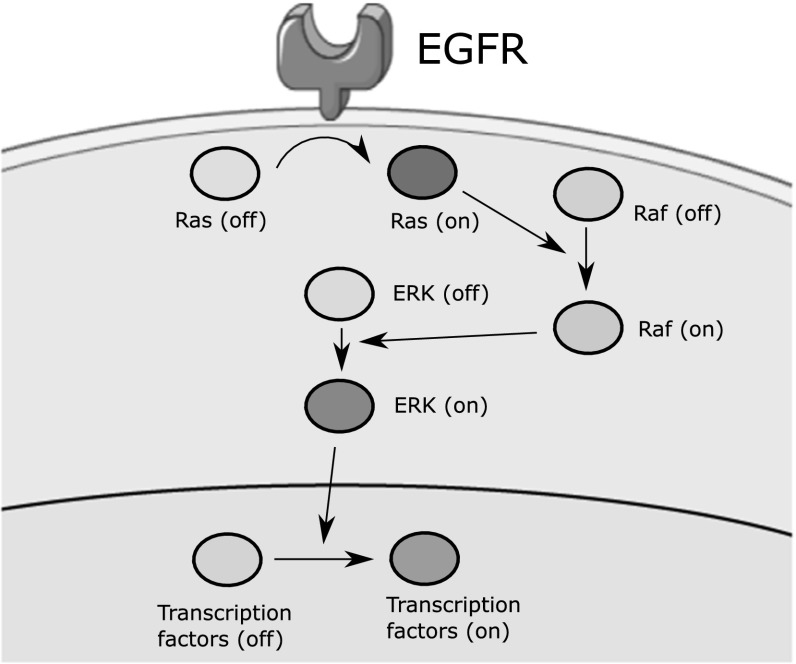
Schematic representation of the part of the EGFR/ERK pathway described by the model

### Intracellular Regulation

To capture the effects of mutational alterations on the fate of each cell, we develop a submodel of intracellular regulation via EGFR/ERK signalling. We use the Brownian Dynamics method to implement the model in order to preserve the spatial and stochastic effects underlying this process. A snapshot of the structure of an individual cell is presented in Fig. 2b.

*Modelling the EGFR/ERK signalling pathway*

EGFR/ERK signalling begins when EGFRs form G-protein binding domains upon their activation by EGF particles. These domains activate the neighbouring Ras proteins which results in the induction of the Ras/Raf/MEK/ERK cascade. The part of the EGFR/ERK pathway described by the model is shown in Fig. 3. We consider the following reactions:

Ras activation by G-protein binding sites (receptors) and its inactivation

$$\begin{aligned} \mathrm{Ras}^\mathrm{off} + \mathrm{EGFR} \rightleftharpoons \mathrm{Ras}^\mathrm{on} \end{aligned}$$

Raf activation by active Ras

$$\begin{aligned} \mathrm{Raf}^\mathrm{off} + \mathrm{Ras}^\mathrm{on} \longrightarrow \mathrm{Raf}^\mathrm{on} + \mathrm{Ras}^\mathrm{on} \end{aligned}$$

Raf switching off

$$\begin{aligned} \mathrm{Raf}^\mathrm{on} \longrightarrow \mathrm{Raf}^\mathrm{off} \end{aligned}$$

ERK activation by active Raf

$$\begin{aligned} \mathrm{ERK}^\mathrm{off} + \mathrm{Raf}^\mathrm{on} \longrightarrow \mathrm{ERK}^\mathrm{on} + \mathrm{Raf}^\mathrm{on} \end{aligned}$$

ERK switching off

$$\begin{aligned} \mathrm{ERK}^\mathrm{on} \longrightarrow \mathrm{ERK}^\mathrm{off} \end{aligned}$$

Transcription factors (TF) activation by active ERK

$$\begin{aligned} \mathrm{TF}^\mathrm{off} + \mathrm{ERK}^\mathrm{on} \longrightarrow \mathrm{TF}^\mathrm{on} + \mathrm{ERK}^\mathrm{on} \end{aligned}$$

TF inactivation

$$\begin{aligned} \mathrm{TF}^\mathrm{on} \longrightarrow \mathrm{TF}^\mathrm{off} \end{aligned}$$

*Brownian Dynamics Simulation*

We implement the previously described model of EGFR/ERK signalling using the BD method. Molecules in the cytoplasm are modelled as hard spheres diffusing according to Brownian motion. Each molecule has a reaction radius $r_\mathrm{BD}$. If two reactive molecules overlap, the corresponding reaction fires. The activation duration of proteins is sampled from an exponential distribution. Acquired mutations in the Ras and Raf proteins reduce their corresponding inactivation rates. We consider that intracellular proteins do not degrade during the lifetime of the cell. This assumption was considered because the half-life time of these proteins is about 24 h which corresponds to the average lifetime of the cell Ramalho et al. (2002).

We suppose that each cell contains a total of 610 particles. Proteins are initialized to be either Ras, Raf, ERK or TF in the ‘off’ state. Ras and Raf molecules can diffuse in the cytoplasm, while TFs can diffuse in the nucleus. ERK proteins are initially present in the cytoplasm but can also diffuse in the nucleus upon their activation. We consider that particles are reflected when they cross the boundary of their respective diffusion domain inside the cell. The number of molecular species in the model are chosen in a way to reduce the computational cost and maintain the consistency with their corresponding copy numbers in biomedical literature (Table 1).

**Table 1:** The numbers and diffusion domains of molecular species involved in the model

Molecular species	Number in the model	Copy number in the experiments	Diffusion domain
Ras	200	400,000 (estimated from Orton et al. (2009))	Cytoplasm
Raf	60	120,000 (estimated from Orton et al. (2009))	Cytoplasm
ERK	300	600,000 (estimated from Orton et al. (2009))	Whole cell
TF	50	100,000 (estimated from Biggin (2011))	Nucleus

The extracellular and intracellular molecules are propagated using an operator split approach. We choose a time step $\Delta t$, propagate the extracellular molecules, then the intracellular molecules, and finally the action of the cells. At the end of the time step, the system is synchronized.

There exist a variety of microscale simulators. Examples are Smoldyn (Andrews and Bray 2004), MCell (De Schutter 2000), and eGFRD (van Zon and Ten Wolde 2005). The former two implement similar fixed time step BD schemes, while the latter, eGFRD, is more detailed and accurate. To simplify the integration with the cell mechanics model, we implemented a BD solver specific to the model that we study in this work. Also, using a more detailed and accurate microscale method, such as eGFRD, would make the simulations prohibitively expensive, and would not be warranted given the resolution of the model.

Simulations can be significantly speeded up compared to a naive BD scheme by considering that molecules in the extracellular space are simulated independently from molecules in the intracellular space. In addition, we do not account for interactions between non-reactive molecules. Finally, we use a relatively large time step, as, in the present study, the qualitative behaviour of the system is more important than the quantitative.

**Fig. 4:**
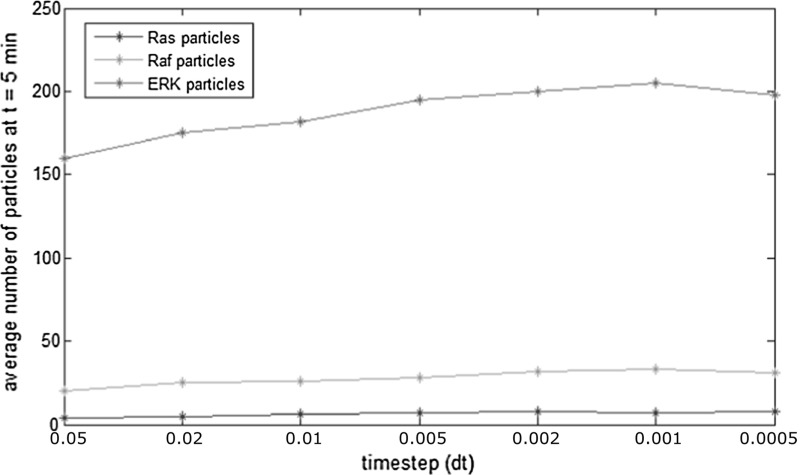
Convergence study of Brownian Dynamics simulations of the intracellular regulation in the absence of mutational changes. The numbers of three molecular species are computed after 30 min of the activation of an EGFR. The results are averaged over ten simulations. We observe that the mean copy number of the species does not change significantly for time steps smaller than 0.005 min

Still, to obtain sufficient accuracy we need to ensure that the time step is not too large. As we have no analytic solution to compare to, we simulate the system with decreasing time step until there is no significant change in mean behaviour. In Fig. 4, we see that for time steps smaller than approximately 0.005 min, there is no significant difference in the average copy number of species. Still, it is possible to observe small fluctuations below this value due to the stochasticities in the model. The values of physiological parameters for the corresponding simulation are given in Table 2.

### A Cell-Based Model of Tumour Growth Mechanics

We use a cell-based approach to describe the development of the tumour. Each cell is represented as an elastic sphere with an incompressible inner part which corresponds to its nucleus. The number of intracellular TFs determines the fate of each cell. If this number exceeds a certain threshold $N_\mathrm{TF}^*$ at the end of the G1-phase, then the cell grows and divide by the end of its cell cycle. Otherwise, it dies by apoptosis. When the cell decides to divide, its size increases linearly until it reaches the double of its initial volume. After the division, two daughter cells appear and the direction of the axis connecting their centres is chosen randomly. The increase in the volume of the mother cell ensures that the daughter cells will not overlap with its neighbours at the moment of their apparition. The daughter cells are half the size of the mother cell at the moment of division. We consider that each daughter cell inherits half of the intracellular proteins of the mother cell. However, their coordinates are reinitialized randomly to account for the dilution effects at the moment of division. The expansion of the tumour is driven by the mechanical interactions between cells resulting from their growth and divisions. We set the cell cycle of all cells to 24 h with a random perturbation that is sampled uniformly from the interval $[-\,3h, 3 h]$. We describe the motion of individual cells as follows using Newton’s law of dynamics:

1 $$\begin{aligned} m {\ddot{x}}_i + m \mu {\dot{x}}_i - \sum _{j \ne i} f_{ij} = 0, \end{aligned}$$

where *m* is the mass of the particle and $\mu$ is the friction factor due to contact with the surrounding medium. The repulsive force between two cells is given explicitly by:

$$\begin{aligned} f_{ij} = \left\{ \begin{array}{ccc} K \frac{h_0 - h_{ij}}{h_{ij} - (h_0-h_1)} &{},&{} h_0-h_i< h_{ij} < h_0 \\ 0 &{},&{} h_{ij} \ge h_0 \end{array} \right. , \end{aligned}$$

where $h_{ij}$ is the distance between the centres of the two cells *i* and *j*, $h_0$ is the sum of their radii, *K* is a positive parameter, and $h_1$ is the sum of the incompressible part of each cell. The force between the particles tends to infinity if $h_{ij}$ decreases to $h_0 - h_1$. The same modelling method was previously used to describe the biomechanics of multicellular systems such as erythropoiesis (Bouchnita et al. 2016b), multiple myeloma (Bouchnita et al. 2016a, 2017a), and the immune-response (Bouchnita et al. 2017b, c). This cell-based model describes the biomechanics of tumour growth in environments with different Reynolds numbers. It can be reduced to a first-order equation by assuming that the inertial term is extremely small in comparison with the dissipative term (Galle et al. 2005; Ghaffarizadeh et al. 2018; Letort et al. 2018; Macklin et al. 2012; Pitt-Francis et al. 2009). This assumption is especially valid for tumours that grow in microenvironments with a low Reynolds number. In our model, we keep the inertia term because we would like to simulate tumour development in different locations of the body.

### Sources of Noise in the Model

There exist several sources of noise in the model which affect the regulation of individual cells and subsequently the development of the tumour. Therefore, the model can be used to explore the effects of stochastic noise on the organization of multicellular systems. The sources of noise can be divided into intrinsic and extrinsic cellular noise. Some of the main sources of intrinsic noise are:

1. the distribution of G-protein binding sites on the cell membrane;
2. the stochastic residence time of EGFRs;
3. the low copy number of intracellular molecular species.

Furthermore, the framework models several sources of extrinsic noise, such as:

1. the relatively low number of EGF particles and their random introduction to the domain as well as stochasticity in their degradation time;
2. the fluctuations in the cell cycle;
3. the low number of cells per simulation;
4. the dilution of proteins at the moment of cell division.

## Results

### The Effect of Mutational Alterations in the EGFR/ERK on the Intracellular Regulation of Cells

We illustrate the performance of the model by investigating the effect of cancerous mutations in the EGFR/ERK pathway on the regulation of individual cells as described in a previous work (Brown et al. 2004). This previous study quantified the impact of the K-Ras and B-Raf mutations on the activation of ERK proteins. To achieve this, a validated ODE model was developed and used to describe the case where an active receptor stimulates the EGFR/ERK pathway. The receptor is considered to be activated during the first 8 min. It becomes inactive after this time. As a result, the activation of ERK proteins in the absence of mutations is transient in the normal case. However, when there are additional K-Ras or B-Raf mutations, the observed ERK activation becomes constitutive. The same behaviour was observed in another study on the intracellular activity of ERK (Orton et al. 2009).

We calibrate our model for one cell to reproduce these findings. We consider a cell consisting of intracellular proteins all initially in the ‘off’ state and we set the number of initially active receptor clusters to one. We consider that this receptor becomes inactive after 8 min which is equal to the average of the waiting time of EGFR inactivation. First, we only consider the dynamics of the EGFR/ERK pathway in normal conditions. Then, we simulate the intracellular regulation dynamics when acquired K-Ras and B-Raf mutations upregulate the activity of ERK. These mutations decrease the inactivation rates of the corresponding proteins.

**Fig. 5:**
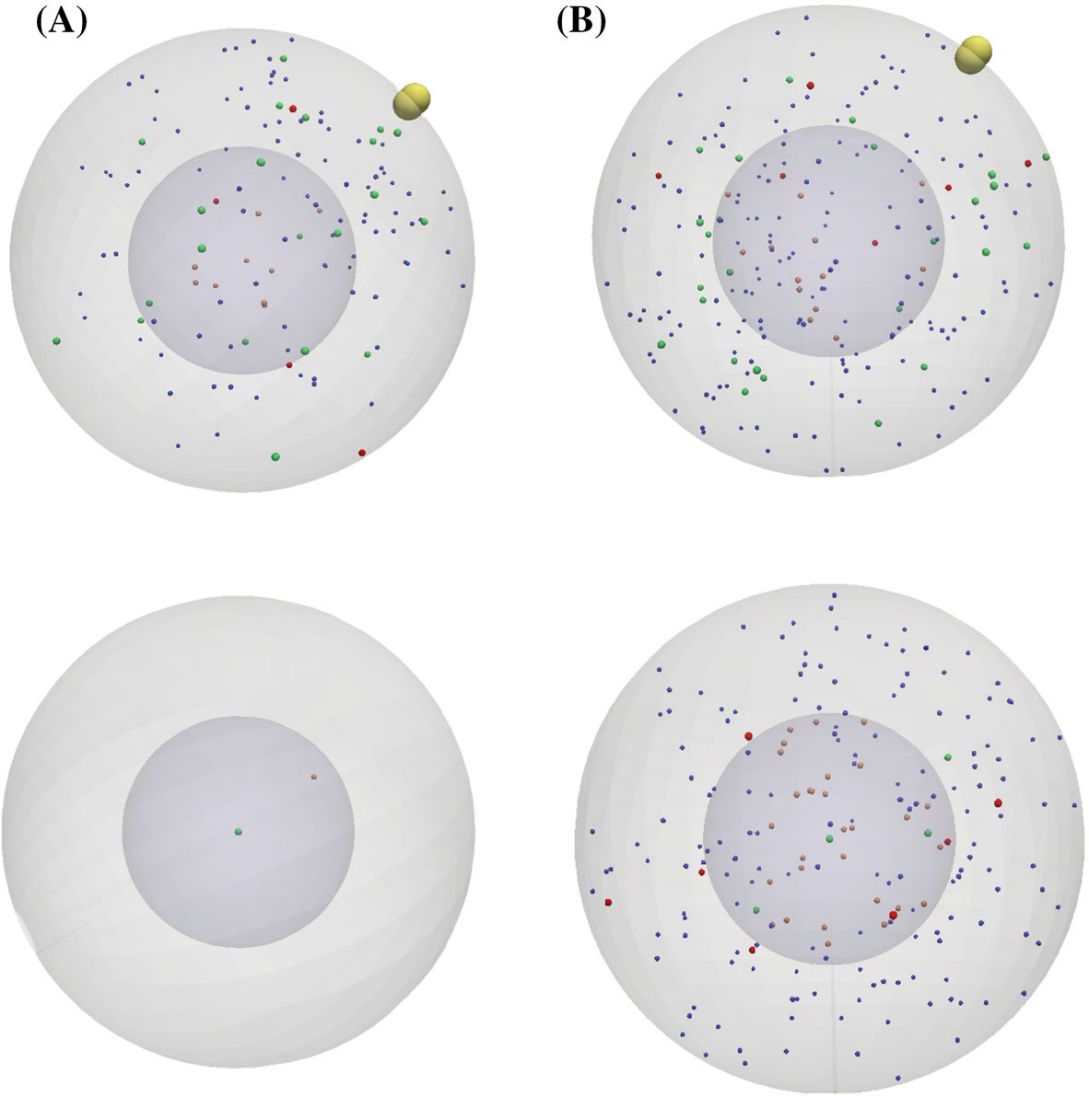
Snapshots of numerical simulations for single-cell regulation at $t = 12$ h (top) and $t = 24$ h (bottom). The intracellular regulation is shown for a cell in the normal case (left), and with an acquired K-Ras mutation (right)

Numerical simulations of intracellular regulation quantify the effects of the acquired K-Ras and B-Ras mutations in upregulating the activity of ERK and TFs. The number of active intracellular proteins is clearly higher when the K-Ras mutation is considered as shown in Fig. 5.

**Fig. 6:**
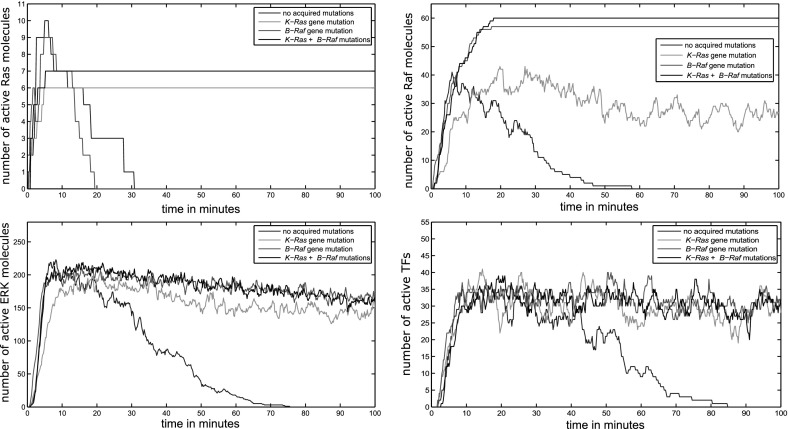
The effects of acquired mutations on the numbers of active proteins during a simulation of intracellular regulation. The represented proteins are Ras (top, left), Raf (top, right), ERK (bottom, left) and transcription factors (bottom, right)

Under normal conditions, the level of active TF is upregulated during the first 40 min of the simulation time. Then, it decreases until it reaches zero. When an acquired K-Ras mutation is considered, it increases the activation time of Ras molecules which results in a prolonged activation of TFs. At the same time, the numbers of Ras, Raf, and ERK follow the same pattern. In other words, they increase and keep fluctuating around a constant value. When the B-Raf mutational alteration is considered, the observed activation of TFs is also constitutive in a similar way to the effect of K-Ras. This equal effect of the K-Ras and B-Raf is in good agreement with a previous study (Orton et al. 2009). Our model predicts that the combined effect of K-Ras and B-Raf mutations on the activation of TFs is closely similar to their separate effects. The individual and combined effects of these mutations on intracellular regulation are illustrated in Fig. 6.

### The Distance Between Simultaneously Active EGFRs Affects the Level of EGFR/ERK Signalling

The activation of EGFRs results in the apparition of G-protein binding sites at the inner surface of the cell membrane. These sites stimulate the EGFR/ERK signalling pathway which promotes the survival and proliferation of cells. Non-spatial models can be used to quantify the effects of EGFR–ligand binding on the intracellular regulation of cells. However, these models do not consider the spatial distribution of EGFRs at the surface of the cell and its effect on cellular regulation. Our model can describe the effect of the spatial distribution of receptors on the intracellular regulation of cells.

**Fig. 7:**
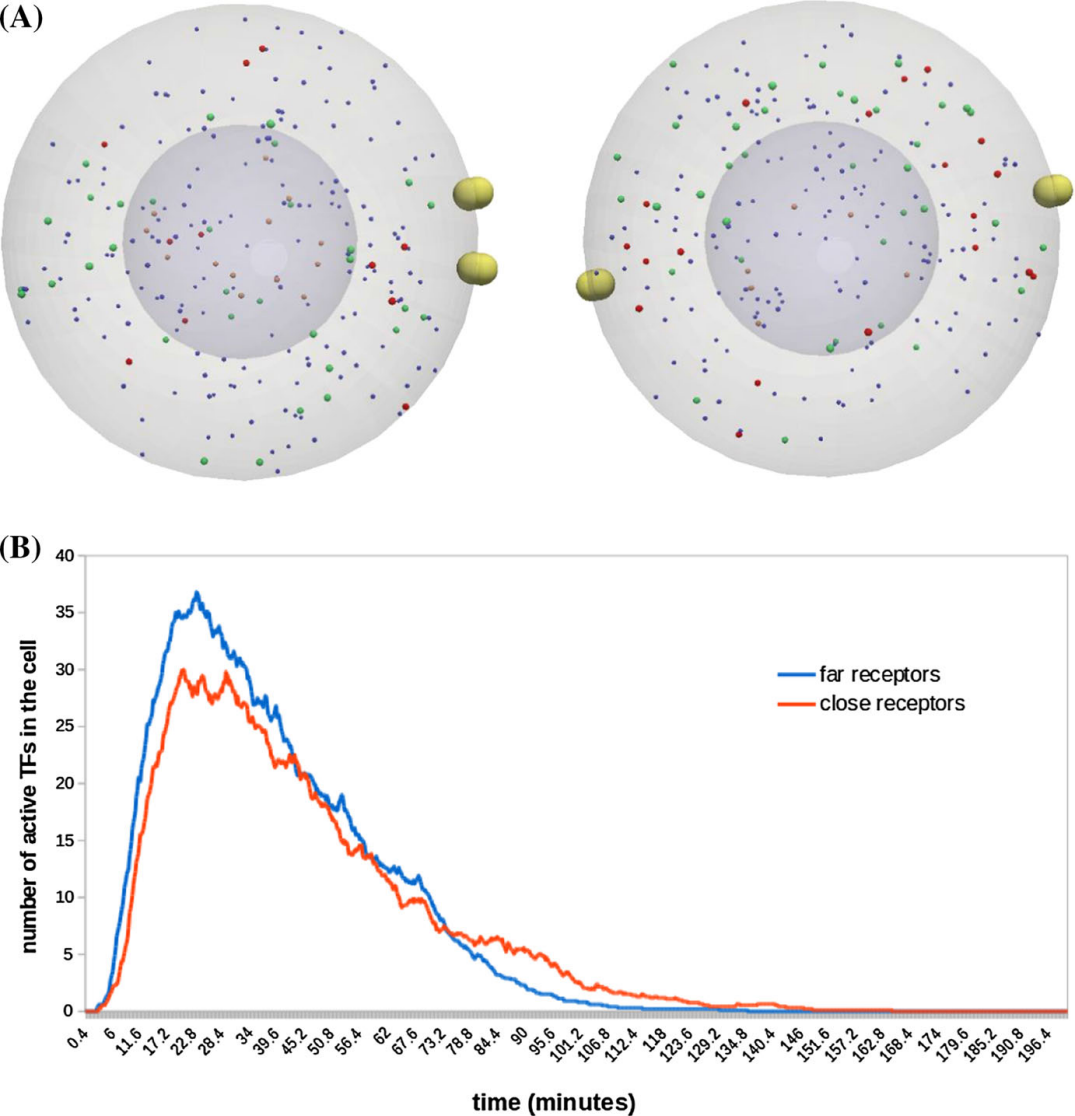
The distribution of active EGFR clusters affects the intracellular regulation of cells. **a** We consider two cells with two active receptors at different configurations. These receptors are close to each other in one (left) and far in the other (right). **b** The averaged numbers of TFs in twenty-five simulations show that expression of TFs remains higher for a longer time in the cell where receptors are located at the opposite poles of the cell

We consider two cases reflecting different distributions of active receptor clusters. In both scenarios, we consider two active receptors but with different relative positions. In the first case, the two receptors are close to each other, whereas in the second case, they are located at opposite poles of the cell (Fig. 7a). The average level of TF activation in both cases is shown in Fig. 7b. We observe that the number of active TFs reaches a higher value in cases where the receptors are far from each other. This is probably because the corresponding G-protein binding sites are exposed to more inactive Ras proteins than when the receptors are close to each other. In other words, the Ras proteins that reach one G-protein site could be already activated by the other G-protein site when the two receptors are close to each other. This situation rarely occurs when active receptors are on the opposite poles of the cell.

### The Effect of EGFR Overexpression on Tumourigenesis

**Fig. 8:**
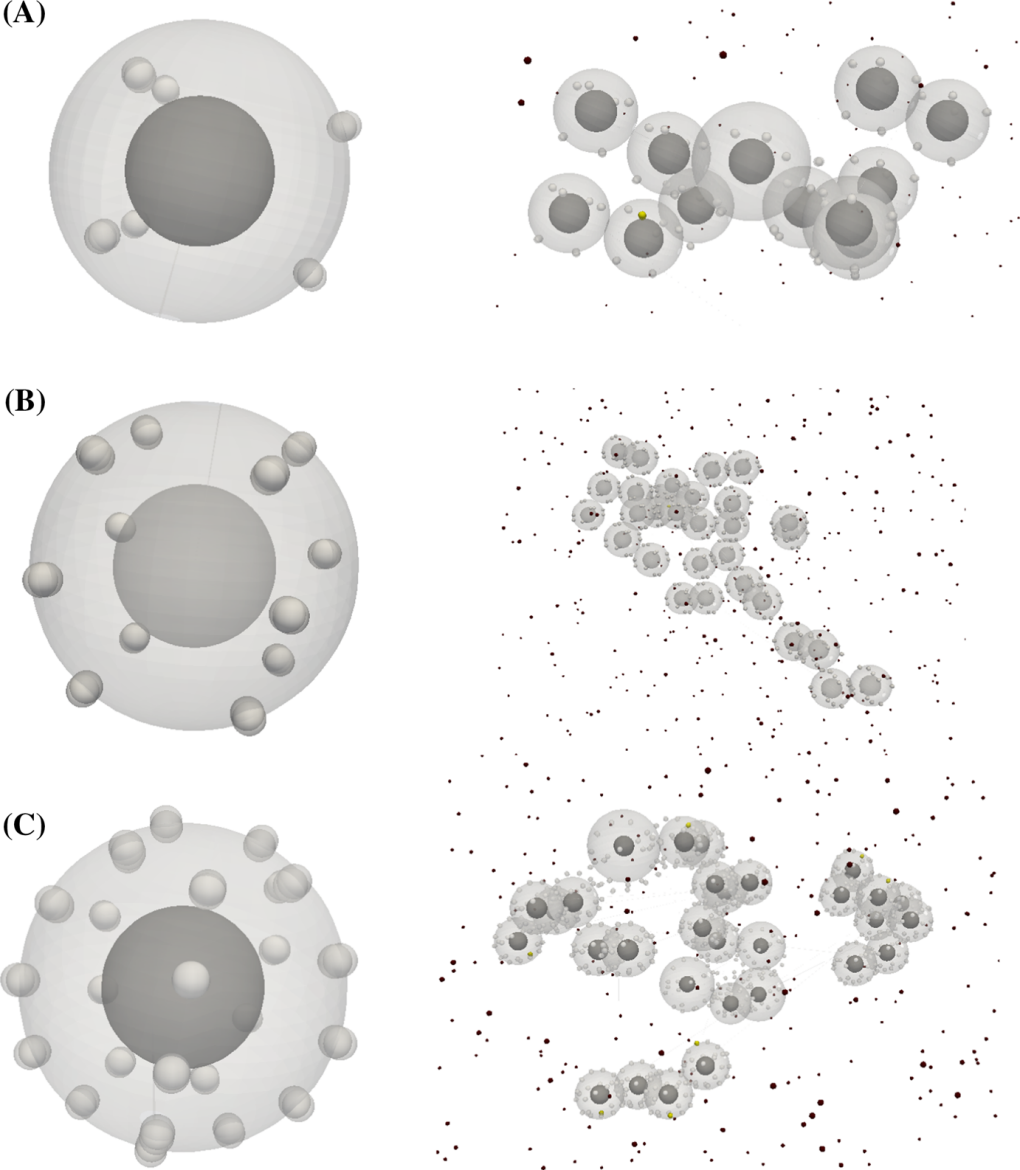
Three configurations of EGFR cluster distribution (left) and the resulting tumour after 2 weeks (right). These configurations consist of 6 (**a**), 12 (**b**), and 20 receptor clusters (**c**) uniformly distributed on the surface of the cell

Acquired mutations in the EGFR gene can result in the overexpression of EGFRs at the surface of the cell. As a result, the cell is able to bind with a higher number of EGF ligands which increases the level of EGFR/ERK signalling. To quantify the effect of the EGFR number on the dynamics of tumour growth, we consider tumours which consist of cells with three distinct configurations of EGFR clusters. These three configurations are shown in Fig. 8, left, and consist of 6, 12, and 24 EGFRs. We run three numerical simulations starting with one tumour-initiating cell at the centre of the computational domain. We consider that the cells do not harbour any other mutations than the one in the EGFR gene.

**Fig. 9:**
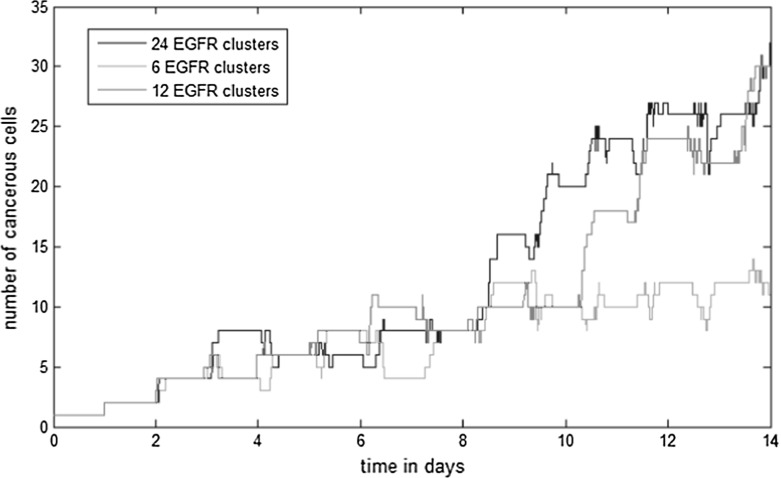
The number of cells in the tumour over time as a function of the number of expressed EGFRs on their surface

The organization of the tumour spheroid model was observed during the three simulations (Fig. 8, right). This organization characterizes in vitro tumours and consists of cells surrounding a necrotic core (Zanoni et al. 2016). This is because EGF particles bind to the outer tumour cells and cannot penetrate to the inside of the tumour. Numerical simulations predict a significantly higher growth rate in the two cases where the number of EGFRs per cell is equal to 12 and 24 than in the case where their number is 6 (Fig. 9). Furthermore, simulations reveal that the growth rate is almost the same when the number of EGFRs is 12 and 24. This indicates that the signalling becomes saturated when the number of EGFRs per cell exceeds 12.

### The Effect of Mutations on the Rate of Tumour Growth

**Fig. 10:**
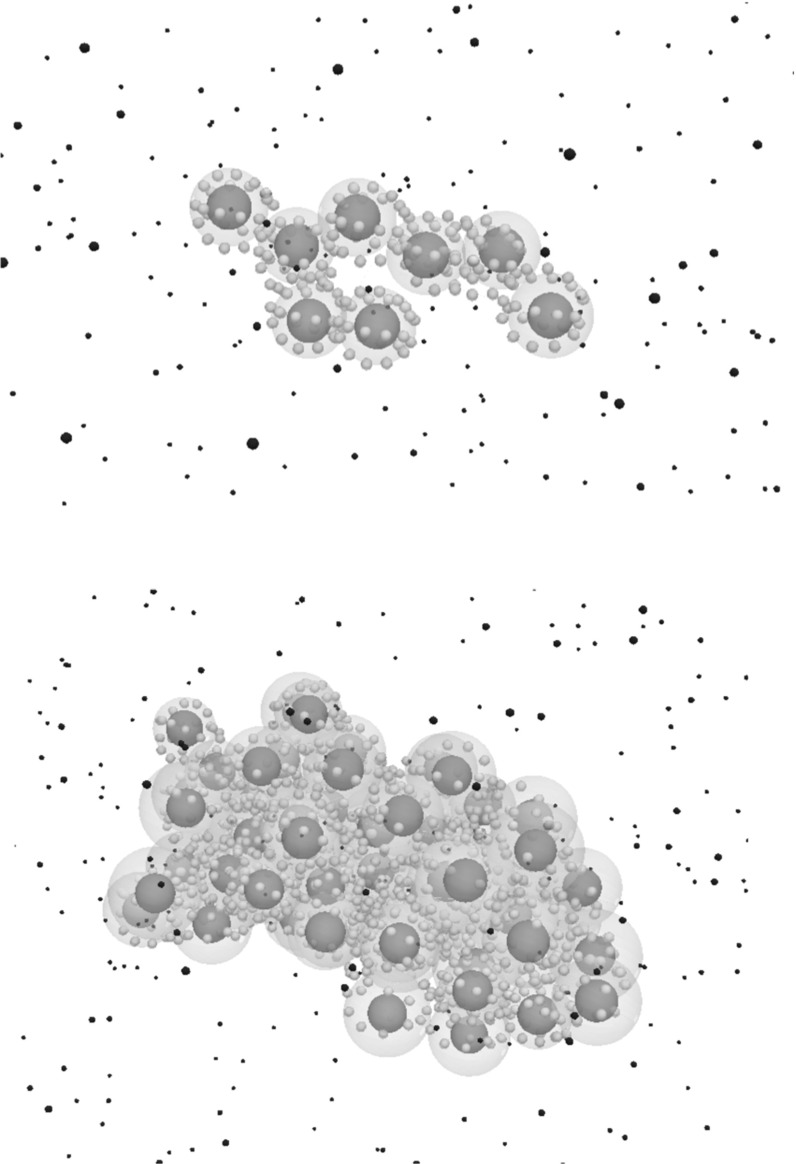
Snapshots of a numerical simulation describing the early stage of a tumour driven by an acquired K-Ras mutation after 1 week (top) and 2 weeks (bottom) of the mutation acquisition

Mutations disrupt the normal functioning of EGFR/ERK signalling which increases the growth rate of the tumour. We simulate different scenarios where we introduce a tumour-initiating cell with different mutational alterations in each case. These changes include the previously described K-Ras and B-Raf alterations in addition to the EGFR mutation where the overexpression of EGFR receptors can be observed. Snapshots of a numerical simulation of a tumour driven by a K-Ras mutation is shown in Fig. 10.

**Fig. 11:**
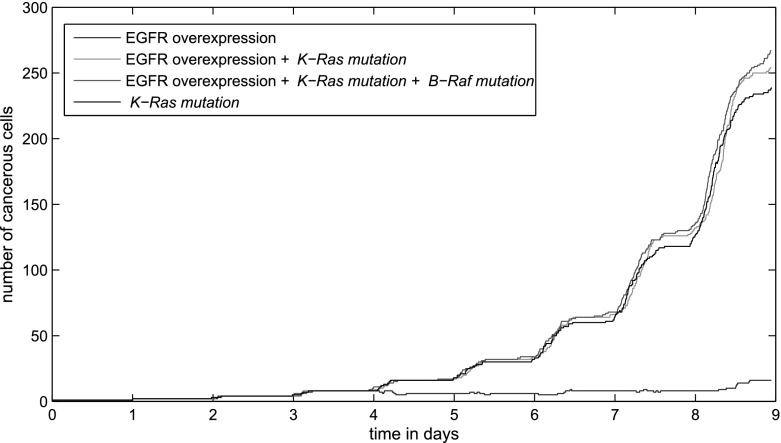
The evolution of tumour populations as a function of genetic changes in cells caused by mutations

According to our simulation results, EGFR overexpression is not able to significantly increase the growth rate of the tumour in the absence of other mutations. In this case, the number of cells grows slowly. When additional acquired mutations are considered, the characteristic exponential growth of the tumour becomes clear. In this context, the K-Ras and B-Raf mutations have approximately the same effect on the development of the tumour. This is because these mutations have the same effects on the activity of ERK and the subsequent activation of TFs as shown in Sect. 3.1. When both the K-Ras and EGFR mutations are considered, a slight growth in the population of cells can be observed. We have plotted the population of cells over time for tumours driven by different mutations in Fig. 11.

## Discussion

The main purpose of this work was to develop a high-fidelity model and simulation framework capable of demonstrating that detailed spatial stochastic aspects of gene regulation can interplay with cell mechanics in the early stages of tumour initiation. Even with state-of-the-art methodology, such simulations become highly computationally demanding. To make simulations feasible, simplifications had to be introduced in the Brownian Dynamics particle simulations to reduce the computational time. These simplifications include the reduction in the number of proteins and the simplification of reaction models below the physiologically observed copy numbers of the precise proteins in the network that we consider. This means precise quantitative predictions are out of scope of the present model, and in particular, it could risk to exaggerate the relative protein noise levels for the particular intracellular regulation network in this paper. However, the framework serves the purpose of qualitatively exploring how detailed spatial stochastic kinetics and mutational alterations in single cells can influence phenomenological predictions. Our numerical experiments clearly illustrate a number of processes that are possible to study when stochastic single-cell kinetics is incorporated in a cell-based framework. Encouraged by these predictions, we are actively pursuing a) multiscale techniques to reduce the cost of single-cell simulations to extend the range of protein copy numbers that can be feasibly simulated, and b) massively parallel simulations in modern distributed and cloud environments using the Orchestral toolkit (Coulier and Hellander 2018). Indeed, an efficient computer implementation is necessary in order to perform numerical simulations of complex tumour models in an affordable CPU time (Szymańska et al. 2018). Therefore, we wrote our code in a modular way and in the object-oriented programming (OOP) style. The components of the model, such as cells, receptors, cytokines, and proteins, were introduced as classes in the code. The computational space was divided into boxes to efficiently simulate the interactions between proteins and cells. To further reduce the computational cost of BD simulations, we intend to implement a tau-leaping method in the future (Gillespie 2001).

The reported multiscale model represents a novel tool to study the dynamics of tumour initiation under various conditions. The model is organized in a multiscale architecture which integrates several modules for the underlying physiological processes ranging from the intracellular dynamics of individual cells and the EGFR–ligands interactions to the biomechanics of multicellular systems. The performance of our framework was tested using a bottom-top approach. We began by investigating the effects of mutational alterations on the intracellular dynamics of individual cells. The predictions of the model qualitatively agree with the findings reported in earlier work where the kinetics of the EGFR/EERK pathway were simulated (Brown et al. 2004; Orton et al. 2009). The effects of the spatial distribution of active receptors on the regulation of individual cells were also explored using numerical simulations. Then, we have quantified the individual and combined effects of mutations on the dynamics of tumour initiation. Despite the low number of considered cells and the various sources of stochastic noise in the model, we were able to observe the apparition of the documented morphology of 3D tumour spheroids (Zanoni et al. 2016). The model simulates tumour growth in the absence of the surrounding healthy cells. This assumption was considered in order to reduce the computational cost. We expect that surrounding cells will exert mechanical force which would result in the contraction of the tumour and the minimization the gaps between cells. The present modelling framework can be used to investigate the effects of various biophysical parameters on the growth rate of the tumour such as the size of the cell and diffusion coefficients. We expect that increasing the cell size or decreasing the diffusion coefficients would reduce the occurrence of reactions. As a result, the activation of TFs would diminish which would, in turn, decrease the chances of cell proliferation.

However, the model introduced in this study presents few limitations. The main one concerns the high computational cost due to the complex and physiologically relevant modelling of individual cells. While it is possible to describe the growth of tumours with a low number of cells, large-scale simulations become very expensive for the computational power a normal computer. Therefore, the number of proteins involved in the regulation of cells was reduced in order to decrease the computational cost and make numerical simulations affordable. An important assumption that was considered in this model concerns the shape of receptors. These sites are considered as two clamped spheres, whereas it is probably better to represent them as cylinders. This simplifying hypothesis was considered because of the simplicity in treating spherical geometries. Other simplifications considered in the framework concern the EGFR/ERK pathway. We have captured the essential functioning of this cascade by considering only three proteins: Ras, Raf, ERK, whereas in reality, it contains many more components and feedback mechanisms (Orton et al. 2009; Schoeberl et al. 2002).

Overall, the multiscale modelling framework presented in this work is most appropriate for studies in systems biology. While most multiscale models of tumour growth focus on higher-level physiology and interactions, mutational alterations directly affect the functioning of individual cells. Hence, it is important to accurately capture such effects on the cell-scale level in order to properly simulate the development of tumours. Among the features of the presented framework is the possibility to integrate different scale-specific data extracted from the biomedical literature. In this regard, it is possible to integrate the data obtained by a broad range of acquisition techniques such as flow cytometry, nano-imaging, and transcriptome sequencing. The framework also considers the various perturbations and fluctuations that shape the biological processes at different scales which has the potential to affect the global behaviour of the system. In a forthcoming study, we will apply our framework to assess the efficiency of EGFR inhibitors in the treatment of different types of cancer. In particular, we would like to study how biological noises affect the efficacy of these treatments.

## Appendix

### A: Parameter Values

**Table 2:** Values of physiological parameters

Parameter	Value in the model	Physiological value	Description
*dt*	0.01 min		Time step
$D_\mathrm{EGF}$	6 $\upmu \mathrm{m}^2 \mathrm{min}^{-1}$	6 $\upmu \mathrm{m}^2~\mathrm{min}^{-1}$	EGF diffusion coefficient (Dale et al. 1994)
$\gamma _\mathrm{EGF}$	0.0115 min$^{-1}$	$\ln (2)/60$ min$^{-1}$	EGF degradation time (Dale et al. 1994)
*K*	1000 $\mathrm{kg}~\upmu \mathrm{m}^{-1}~\mathrm{min}^{-1}$		Contact force coefficient
Cell characteristics			
$d_\mathrm{cell}$	4 $\upmu \mathrm{m}$		Cell diameter
$d_\mathrm{nuc}$	2 $\upmu \mathrm{m}$		Cell nucleus diameter
$N_\mathrm{TF}^*$	23		Number of TFs for cell division
$t_\mathrm{cell}$	24 $\pm \; 2$ h		Cell cycle
$N_\mathrm{EGF}$	660 mol	$6.6 \times 10^3$ nM	EGF normal concentration (Dale et al. 1994)
$N_\mathrm{EGFR}/\mathrm{cell}$	Variable (6, 12 or 20)	$4 \times 10^5$	Number of EGFR per cell (Schoeberl et al. 2002)
$\gamma _\mathrm{EGFR}$	0.125 min$^{-1}$	$\ln (2)/82.5 \; \mathrm{min}^{-1}$	EGFR/EGF dissociation time (Zhang et al. 2015)
Brownian Dynamics parameters			
$r_\mathrm{BD}$	0.15 $\mu \mathrm{m}$		Reaction radius in BD simulations
$\gamma _\mathrm{Ras}$	$3e^{-2}$ min$^{-1}$		Inactivation rate of active Ras (non-mutated)
$\gamma _\mathrm{Ras}$	$1e^{-9}$ min$^{-1}$		Inactivation rate of active Ras (mutated)
$\gamma _\mathrm{Raf}$	$3e^{-2}$ min$^{-1}$		Inactivation rate of active Raf (non-mutated)
$\gamma _\mathrm{Raf}$	$1e^{-9}$ min$^{-1}$		Inactivation rate of active Raf (mutated)
$\gamma _\mathrm{ERK}$	$1e^{-1}$ min$^{-1}$		Inactivation rate of active ERK
$\gamma _\mathrm{TF}$	$5e^{-2}$ min$^{-1}$		Inactivation rate of active TFs

The values of model parameters for cell characteristics were estimated from the literature. The number of molecules was reduced in order to decrease the computational cost. Brownian Dynamics parameters were fitted to reproduce previous experiments (Orton et al. 2009). This was possible because it takes about 3 min to simulate 100 min of the intracellular regulation of one cell. We began the calibration process by choosing the reaction radius that gives us approximate results. Then, we have fitted the degradation rates to obtain more accuracy and reproduce the experimental results. The values of physiological parameters for a basic simulation are provided in Table 2.

### B: Computer Implementation

The code was written in C++ in the object-oriented programming (OOP) style. The GNU Scientific Library (GSL) was used for the sampling of random variables. The average CPU time of a numerical simulation corresponding to 2 weeks of tumour initiation is 12 h. The visualizations were generated using the software ParaView. The source code is available upon request to the first author.
